# Correction: Exploring the role of epidermal growth factor receptor variant III in meningeal tumors

**DOI:** 10.1371/journal.pone.0323060

**Published:** 2025-04-17

**Authors:** Rashmi Rana, Vaishnavi Rathi, Kirti Chauhan, Kriti Jain, Satnam Singh Chhabra, Rajesh Acharya, Samir Kumar Kalra, Anshul Gupta, Sunila Jain, Nirmal Kumar Ganguly, Dharmendra Kumar Yadav

The Fig 5 Grade II Control panel in [1] is incorrect. The authors have provided an updated Fig 5 presenting the correct Grade II Control panel, and the original data underlying the published results (S1-S4 Files). The authors clarify that the “control” mentioned in Fig 5 represents an unstained sample of the same grade, and they have updated the figure label accordingly. Furthermore, the authors comment that the results in the Fig 5 GRADE I Anti-EGFR vIII were rounded incorrectly. The rounding error has been corrected in the updated figure.

**Fig 5:**
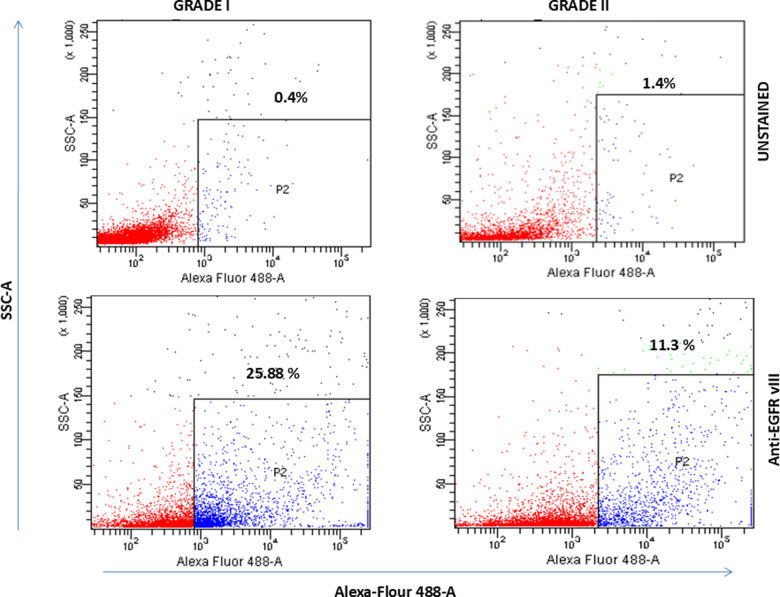
Generation of EGFRvIII− and EGFRvIII+ sublines as determined via FACS. MG Grade I representing 25.88% EGFRvIII (Alexa flour 488) positive cells (SSC vs Alexa Flour 488) while Grade II representing 11.3% EGFRvIII (Alexa flour 488) positive cells (SSC vs Alexa Flour 488). There is about 2 folds decrease in EGFR V3+ cells in grade II as compared to grade I. Unlabelled cells were used as controls in both grade I and grade II.

The updated figure and underlying data were assessed by an independent member of the *PLOS One* Editorial Board, who commented that the updated figure and the underlying data provided by the authors adequately resolve the issues raised with Fig 5.

In addition, there are errors in the Author Contributions listed for this article [1]. The correct contributions are:

**Conceptualization:** Rashmi Rana, Dharmendra Kumar Yadav.

**Data curation:** Rashmi Rana, Vaishnavi Rathi, Kirti Chauhan, Kriti Jain, Satnam Singh Chhabra,

Rajesh Acharya, Samir Kumar Kalra, Anshul Gupta, Sunila Jain.

**Formal analysis:** Rashmi Rana, Dharmendra Kumar Yadav, Vaishnavi Rathi, Kirti Chauhan.

**Funding acquisition:** Rashmi Rana.

**Investigation:** Rashmi Rana, Dharmendra Kumar Yadav.

**Methodology:** Rashmi Rana, Vaishnavi Rathi, Kirti Chauhan.

**Resources:** Nirmal Kumar Ganguly.

**Visualization:** Rashmi Rana, Dharmendra Kumar Yadav.

## Supporting information

S1 File Analysis underlying tables and figures presented in this article. (DOCX)

S2 File Raw FACS data underlying the Fig 5 results. (ZIP)

S3 File Data underlying FACS EGFR VIII positive cells results. (XLSX)

S4 File Data underlying total intensity for EGFR VIII and Ki67 results. (XLS)
