# Supplementary figures and images for: Exploring the role of epidermal growth factor receptor variant III in meningeal tumors
Source: PLoS One. 2021 Sep 28;16(9):e0255133. doi: 10.1371/journal.pone.0255133 (PMC8478197; doi:10.1371/journal.pone.0255133)

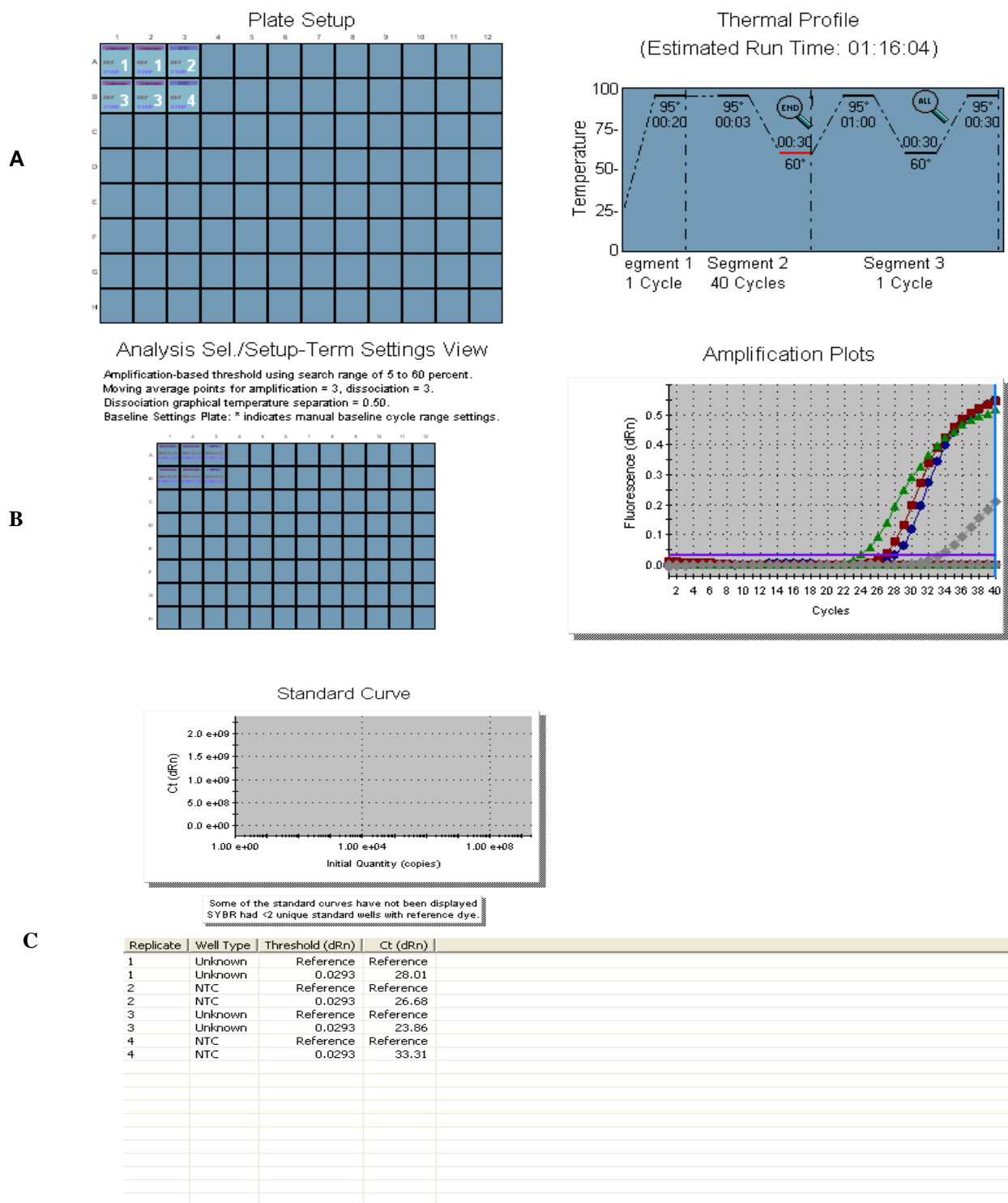

**Figure: S1**

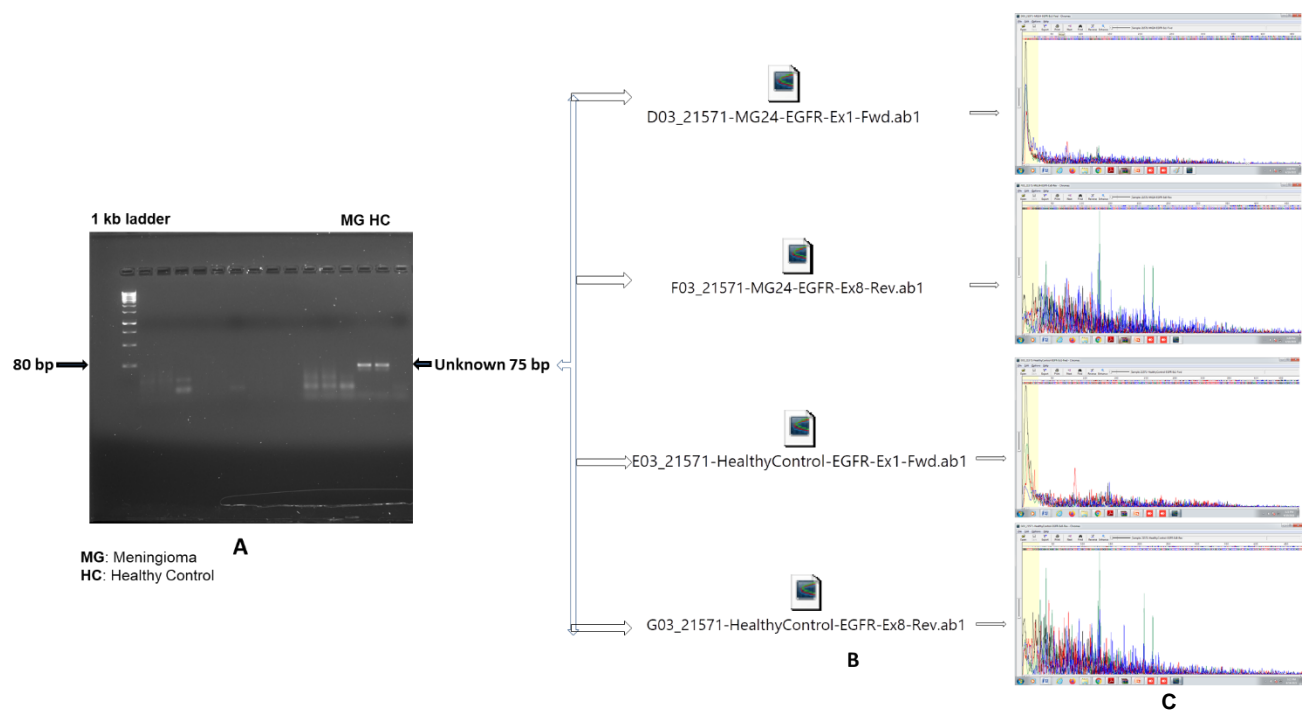

**Figure: S2**

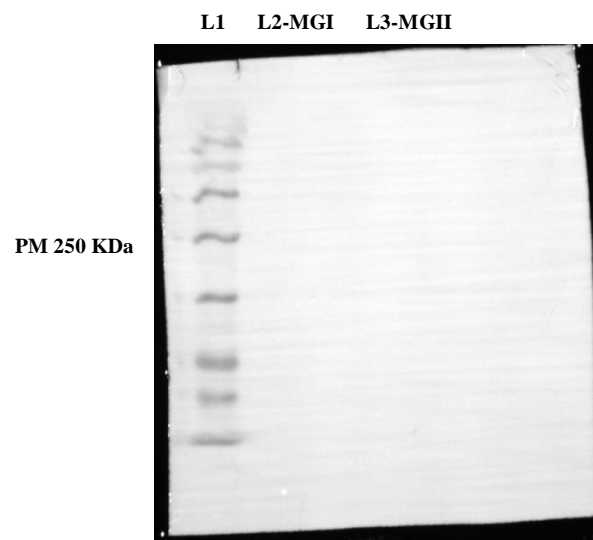

**Figure: S3**

Supplement: S1 Raw images — S1 Fig. EGFR vIII RT-PCR Result (A) Standardized PCR cycle (B) amplification plot showing β-actin (reference material) (C) CT value of assessed samples expressed the beta-actin (reference), result showed that the expression of EFGR vIII was not detected in grade I and grade II meningioma using qRT-PCR. S2 Fig. Screening and mapping of EGFR vIII deletions in MG (A) Design of the RTPCR primers specific for the EGFR vIII mutant. (B) Design of mapping PCR primers corresponding to the EGFR Viii (C) No DNA sequence results seen. S3 Fig. Western blotting result by Anti-EGFR vIII antibody not detected any band of EGFR vIII in meningioma (Grade I & II) (PDF) [file pone.0255133.s001.pdf]
