# Supplementary material for: Correction: Exploring the role of epidermal growth factor receptor variant III in meningeal tumors
Source: PLoS One. 2025 Apr 17;20(4):e0323060. doi: 10.1371/journal.pone.0323060 (PMC12005530; doi:10.1371/journal.pone.0323060)
Supplement: S1 File — (DOCX) [file pone.0323060.s001.docx]

| **Analysis of EFGR VIII intensity/expression**  **MG Grades * EGFR VIII Intensity/Expression Crosstabulation** | | | | | | |
| --- | --- | --- | --- | --- | --- | --- |
|  |  |  | EGFR VIII Intensity/Expression | | | Total |
|  |  |  | 1+ | 2+ | 3+ |  |
| MG Grades | I | Count | 0 | 10 | 24 | 34 |
|  |  | % within MG Grades | .0% | 29.4% | 70.6% | 100.0% |
|  | II | Count | 22 | 0 | 0 | 22 |
|  |  | % within MG Grades | 100.0% | .0% | .0% | 100.0% |
| Total | | Count | 22 | 10 | 24 | 56 |
|  |  | % within MG Grades | 39.3% | 17.9% | 42.9% | 100.0% |

**Table 3 and Figure 4 are made from the above analysis**

| **Chi-Square Tests** | | | |
| --- | --- | --- | --- |
|  | Value | df | Asymp. Sig. (2-sided) |
| Pearson Chi-Square | 56.000^a^ | 2 | .000 |
| Likelihood Ratio | 75.041 | 2 | .000 |
| N of Valid Cases | 56 |  |  |
| a. 1 cells (16.7%) have expected count less than 5. The minimum expected count is 3.93. | | | |

**Frequencies**

**MG Grades = I**

| **Statistics^a^** | | |
| --- | --- | --- |
| AVERAGE |  |  |
| N | Valid | 34 |
|  | Missing | 0 |
| Mean | | 72.81536% |
| Median | | 74.00000% |
| Std. Deviation | | 5.456983% |
| Minimum | | 61.000% |
| Maximum | | 82.000% |
| Percentiles | 25 | 68.75000% |
|  | 50 | 74.00000% |
|  | 75 | 77.00000% |
| a. MG Grades = I | |  |

**MG Grades = II**

| **Statistics^a^** | | |
| --- | --- | --- |
| AVERAGE |  |  |
| N | Valid | 22 |
|  | Missing | 0 |
| Mean | | 35.53864% |
| Median | | 36.50000% |
| Std. Deviation | | 9.951046% |
| Minimum | | .350% |
| Maximum | | 46.000% |
| Percentiles | 25 | 32.50000% |
|  | 50 | 36.50000% |
|  | 75 | 42.12500% |
| a. MG Grades = II | |  |

**Frequencies**

[DataSet1]

**EGFR VIII  Intensity/Expression  = 1+**

| **Statistics^a^** | | |
| --- | --- | --- |
| AVERAGE |  |  |
| N | Valid | 22 |
|  | Missing | 0 |
| Mean | | 35.53864% |
| Median | | 36.50000% |
| Std. Deviation | | 9.951046% |
| Minimum | | .350% |
| Maximum | | 46.000% |
| Percentiles | 25 | 32.50000% |
|  | 50 | 36.50000% |
|  | 75 | 42.12500% |
| a. EGFR VIII Intensity/Expression = 1+ | | |

**EGFR VIII  Intensity/Expression  = 2+**

| **Statistics^a^** | | |
| --- | --- | --- |
| AVERAGE |  |  |
| N | Valid | 10 |
|  | Missing | 0 |
| Mean | | 66.10000% |
| Median | | 67.00000% |
| Std. Deviation | | 4.040077% |
| Minimum | | 61.000% |
| Maximum | | 72.000% |
| Percentiles | 25 | 62.50000% |
|  | 50 | 67.00000% |
|  | 75 | 69.00000% |
| a. EGFR VIII Intensity/Expression = 2+ | | |

**EGFR VIII  Intensity/Expression  = 3+**

| **Statistics^a^** | | |
| --- | --- | --- |
| AVERAGE |  |  |
| N | Valid | 24 |
|  | Missing | 0 |
| Mean | | 75.61343% |
| Median | | 75.00000% |
| Std. Deviation | | 2.926157% |
| Minimum | | 69.000% |
| Maximum | | 82.000% |
| Percentiles | 25 | 74.00000% |
|  | 50 | 75.00000% |
|  | 75 | 77.50000% |
| a. EGFR VIII Intensity/Expression = 3+ | | |

**Analysis of FACS EGFR VIII positive cells and Ki67 positive cells**

| **Statistics** | | | | | | |
| --- | --- | --- | --- | --- | --- | --- |
|  |  | Average | Age | Ki 67 postive | FACS EGFR VIII positive cells (%) | Tumor_VOLUME |
| N | Valid | 56 | 56 | 56 | 56 | 56 |
|  | Missing | 0 | 0 | 0 | 0 | 0 |
| Mean | | 58.17093% | 44.59 | 6.2857% | 22.16571% | 16.92776 |
| Std. Deviation | | 19.827739% | 12.232 | 2.75492% | 10.297690% | 14.599671 |
| Minimum | | .350% | 22 | 3.00% | 2.700% | .117 |
| Maximum | | 82.000% | 65 | 14.00% | 50.500% | 62.790 |
| Percentiles | 25 | 40.25000% | 36.00 | 4.0000% | 14.60000% | 5.58825 |
|  | 50 | 67.00000% | 44.00 | 6.0000% | 20.55000% | 13.32850 |
|  | 75 | 74.50000% | 54.50 | 8.0000% | 26.70000% | 25.11600 |

| **(T-test) Group Statistics** | | | | | |
| --- | --- | --- | --- | --- | --- |
|  | MG Grades | N | Mean | Std. Deviation | Std. Error Mean |
| FACS EGFR VIII positive cells (%) | 1 | 34 | 28.16706% | 8.383717% | 1.437796% |
|  | 2 | 22 | 12.89091% | 4.341594% | .925631% |

Table 2 and table 4 are made using the above analysis of FACS EGFR VIII POSITIVE cells and Ki67 positive cells

**Analysis of other parameters**

**Kaplan-Meier**

| **Case Processing Summary** | | | |
| --- | --- | --- | --- |
| Total N | N of Events | Censored | |
|  |  | N | Percent |
| 56 | 6 | 50 | 89.3% |

| **Survival Table** | | | | | | |
| --- | --- | --- | --- | --- | --- | --- |
|  | Time | Status | Cumulative Proportion Surviving at the Time | | N of Cumulative Events | N of Remaining Cases |
|  |  |  | Estimate | Std. Error |  |  |
| 1 | 4.000 | Died | .982 | .018 | 1 | 54 |
| 2 | 4.000 | Survived | . | . | 1 | 53 |
| 3 | 4.000 | Survived | . | . | 1 | 52 |
| 4 | 4.000 | Survived | . | . | 1 | 51 |
| 5 | 5.000 | Survived | . | . | 1 | 50 |
| 6 | 5.000 | Survived | . | . | 1 | 49 |
| 7 | 5.000 | Survived | . | . | 1 | 48 |
| 8 | 5.000 | Survived | . | . | 1 | 47 |
| 9 | 6.000 | Died | .961 | .027 | 2 | 46 |
| 10 | 6.000 | Survived | . | . | 2 | 45 |
| 11 | 6.000 | Survived | . | . | 2 | 44 |
| 12 | 6.000 | Survived | . | . | 2 | 43 |
| 13 | 7.000 | Survived | . | . | 2 | 42 |
| 14 | 7.000 | Survived | . | . | 2 | 41 |
| 15 | 7.000 | Survived | . | . | 2 | 40 |
| 16 | 7.000 | Survived | . | . | 2 | 39 |
| 17 | 8.000 | Died | . | . | 3 | 38 |
| 18 | 8.000 | Died | .912 | .043 | 4 | 37 |
| 19 | 8.000 | Survived | . | . | 4 | 36 |
| 20 | 8.000 | Survived | . | . | 4 | 35 |
| 21 | 9.000 | Survived | . | . | 4 | 34 |
| 22 | 9.000 | Survived | . | . | 4 | 33 |
| 23 | 9.000 | Survived | . | . | 4 | 32 |
| 24 | 9.000 | Survived | . | . | 4 | 31 |
| 25 | 9.000 | Survived | . | . | 4 | 30 |
| 26 | 10.000 | Died | .881 | .051 | 5 | 29 |
| 27 | 10.000 | Survived | . | . | 5 | 28 |
| 28 | 10.000 | Survived | . | . | 5 | 27 |
| 29 | 11.000 | Survived | . | . | 5 | 26 |
| 30 | 11.000 | Survived | . | . | 5 | 25 |
| 31 | 11.000 | Survived | . | . | 5 | 24 |
| 32 | 12.000 | Survived | . | . | 5 | 23 |
| 33 | 12.000 | Survived | . | . | 5 | 22 |
| 34 | 12.000 | Survived | . | . | 5 | 21 |
| 35 | 12.000 | Survived | . | . | 5 | 20 |
| 36 | 13.000 | Survived | . | . | 5 | 19 |
| 37 | 13.000 | Survived | . | . | 5 | 18 |
| 38 | 14.000 | Survived | . | . | 5 | 17 |
| 39 | 14.000 | Survived | . | . | 5 | 16 |
| 40 | 14.000 | Survived | . | . | 5 | 15 |
| 41 | 14.000 | Survived | . | . | 5 | 14 |
| 42 | 15.000 | Survived | . | . | 5 | 13 |
| 43 | 15.000 | Survived | . | . | 5 | 12 |
| 44 | 16.000 | Survived | . | . | 5 | 11 |
| 45 | 16.000 | Survived | . | . | 5 | 10 |
| 46 | 17.000 | Survived | . | . | 5 | 9 |
| 47 | 18.000 | Survived | . | . | 5 | 8 |
| 48 | 18.000 | Survived | . | . | 5 | 7 |
| 49 | 19.000 | Died | .755 | .124 | 6 | 6 |
| 50 | 19.000 | Survived | . | . | 6 | 5 |
| 51 | 19.000 | Survived | . | . | 6 | 4 |
| 52 | 20.000 | Survived | . | . | 6 | 3 |
| 53 | 20.000 | Survived | . | . | 6 | 2 |
| 54 | 21.000 | Survived | . | . | 6 | 1 |
| 55 | 22.000 | Survived | . | . | 6 | 0 |


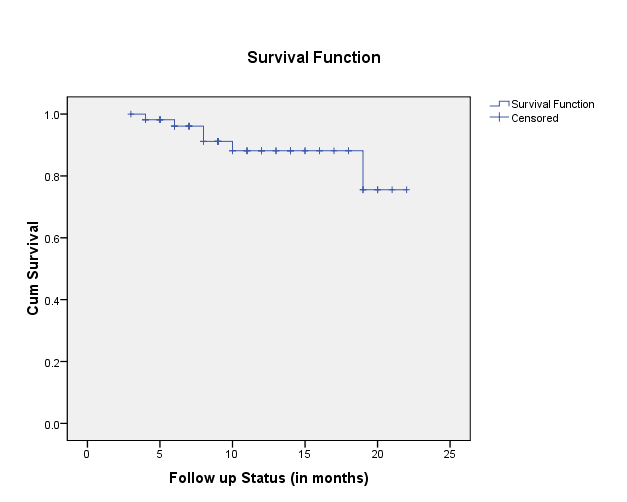


**Crosstabs**

**MG Grades * Seizures**

| **Crosstab** | | | | | |
| --- | --- | --- | --- | --- | --- |
|  |  |  | Seizures | | Total |
|  |  |  | NO | YES |  |
| MG Grades | 1 | Count | 15 | 19 | 34 |
|  |  | % within MG Grades | 44.1% | 55.9% | 100.0% |
|  | 2 | Count | 6 | 16 | 22 |
|  |  | % within MG Grades | 27.3% | 72.7% | 100.0% |
| Total | | Count | 21 | 35 | 56 |
|  |  | % within MG Grades | 37.5% | 62.5% | 100.0% |

| **Chi-Square Tests** | | | | | |
| --- | --- | --- | --- | --- | --- |
|  | Value | df | Asymp. Sig. (2-sided) | Exact Sig. (2-sided) | Exact Sig. (1-sided) |
| Pearson Chi-Square | 1.617^a^ | 1 | .203 |  |  |
| Continuity Correction^b^ | .978 | 1 | .323 |  |  |
| Likelihood Ratio | 1.651 | 1 | .199 |  |  |
| Fisher's Exact Test |  |  |  | .263 | .161 |
| N of Valid Cases^b^ | 56 |  |  |  |  |
| a. 0 cells (.0%) have expected count less than 5. The minimum expected count is 8.25. | | | | | |
| b. Computed only for a 2x2 table | | |  |  |  |

**MG Grades * Headache**

| **Crosstab** | | | | | |
| --- | --- | --- | --- | --- | --- |
|  |  |  | Headache | | Total |
|  |  |  | NO | YES |  |
| MG Grades | 1 | Count | 9 | 25 | 34 |
|  |  | % within MG Grades | 26.5% | 73.5% | 100.0% |
|  | 2 | Count | 4 | 18 | 22 |
|  |  | % within MG Grades | 18.2% | 81.8% | 100.0% |
| Total | | Count | 13 | 43 | 56 |
|  |  | % within MG Grades | 23.2% | 76.8% | 100.0% |

| **Chi-Square Tests** | | | | | |
| --- | --- | --- | --- | --- | --- |
|  | Value | df | Asymp. Sig. (2-sided) | Exact Sig. (2-sided) | Exact Sig. (1-sided) |
| Pearson Chi-Square | .515^a^ | 1 | .473 |  |  |
| Continuity Correction^b^ | .155 | 1 | .694 |  |  |
| Likelihood Ratio | .527 | 1 | .468 |  |  |
| Fisher's Exact Test |  |  |  | .535 | .352 |
| N of Valid Cases^b^ | 56 |  |  |  |  |
| a. 0 cells (.0%) have expected count less than 5. The minimum expected count is 5.11. | | | | | |
| b. Computed only for a 2x2 table | | |  |  |  |

**MG Grades * Vomiting**

| **Crosstab** | | | | | |
| --- | --- | --- | --- | --- | --- |
|  |  |  | Vomiting | | Total |
|  |  |  | NO | YES |  |
| MG Grades | 1 | Count | 27 | 7 | 34 |
|  |  | % within MG Grades | 79.4% | 20.6% | 100.0% |
|  | 2 | Count | 10 | 12 | 22 |
|  |  | % within MG Grades | 45.5% | 54.5% | 100.0% |
| Total | | Count | 37 | 19 | 56 |
|  |  | % within MG Grades | 66.1% | 33.9% | 100.0% |

| **Chi-Square Tests** | | | | | |
| --- | --- | --- | --- | --- | --- |
|  | Value | df | Asymp. Sig. (2-sided) | Exact Sig. (2-sided) | Exact Sig. (1-sided) |
| Pearson Chi-Square | 6.871^a^ | 1 | .009 |  |  |
| Continuity Correction^b^ | 5.439 | 1 | .020 |  |  |
| Likelihood Ratio | 6.852 | 1 | .009 |  |  |
| Fisher's Exact Test |  |  |  | .019 | .010 |
| N of Valid Cases^b^ | 56 |  |  |  |  |
| a. 0 cells (.0%) have expected count less than 5. The minimum expected count is 7.46. | | | | | |
| b. Computed only for a 2x2 table | | |  |  |  |

**Analysis of other parameters**

**Crosstabs**

| **Case Processing Summary** | | | | | | |
| --- | --- | --- | --- | --- | --- | --- |
|  | Cases | | | | | |
|  | Valid | | Missing | | Total | |
|  | N | Percent | N | Percent | N | Percent |
| MG Grades * Neurological_deficit | 56 | 100.0% | 0 | .0% | 56 | 100.0% |
| MG Grades * Extent_of_ resection | 56 | 100.0% | 0 | .0% | 56 | 100.0% |

**MG Grades * Neurological_deficit**

| **Crosstab** | | | | | |
| --- | --- | --- | --- | --- | --- |
|  |  |  | Neurological_deficit | | Total |
|  |  |  | No | yes |  |
| MG Grades | 1 | Count | 23 | 11 | 34 |
|  |  | % within MG Grades | 67.6% | 32.4% | 100.0% |
|  | 2 | Count | 8 | 14 | 22 |
|  |  | % within MG Grades | 36.4% | 63.6% | 100.0% |
| Total | | Count | 31 | 25 | 56 |
|  |  | % within MG Grades | 55.4% | 44.6% | 100.0% |

| **Chi-Square Tests** | | | | | |
| --- | --- | --- | --- | --- | --- |
|  | Value | df | Asymp. Sig. (2-sided) | Exact Sig. (2-sided) | Exact Sig. (1-sided) |
| Pearson Chi-Square | 5.290^a^ | 1 | .021 |  |  |
| Continuity Correction^b^ | 4.099 | 1 | .043 |  |  |
| Likelihood Ratio | 5.341 | 1 | .021 |  |  |
| Fisher's Exact Test |  |  |  | .029 | .021 |
| N of Valid Cases^b^ | 56 |  |  |  |  |
| a. 0 cells (.0%) have expected count less than 5. The minimum expected count is 9.82. | | | | | |
| b. Computed only for a 2x2 table | | |  |  |  |

**MG Grades * Extent_of_ resection**

| **Crosstab** | | | | | |
| --- | --- | --- | --- | --- | --- |
|  |  |  | Extent_of_ resection | | Total |
|  |  |  | (GTR: > 95%) | STR: > 85-95% |  |
| MG Grades | 1 | Count | 33 | 1 | 34 |
|  |  | % within MG Grades | 97.1% | 2.9% | 100.0% |
|  | 2 | Count | 20 | 2 | 22 |
|  |  | % within MG Grades | 90.9% | 9.1% | 100.0% |
| Total | | Count | 53 | 3 | 56 |
|  |  | % within MG Grades | 94.6% | 5.4% | 100.0% |

| **Chi-Square Tests** | | | | | |
| --- | --- | --- | --- | --- | --- |
|  | Value | df | Asymp. Sig. (2-sided) | Exact Sig. (2-sided) | Exact Sig. (1-sided) |
| Pearson Chi-Square | .996^a^ | 1 | .318 |  |  |
| Continuity Correction^b^ | .153 | 1 | .696 |  |  |
| Likelihood Ratio | .970 | 1 | .325 |  |  |
| Fisher's Exact Test |  |  |  | .555 | .339 |
| N of Valid Cases^b^ | 56 |  |  |  |  |
| a. 2 cells (50.0%) have expected count less than 5. The minimum expected count is 1.18. | | | | | |
| b. Computed only for a 2x2 table | | |  |  |  |
